# Supplementary material for: Characterization of Depressive Symptom Trajectories in Women between Childbirth and Diagnosis
Source: J Pers Med. 2022 Mar 28;12(4):538. doi: 10.3390/jpm12040538 (PMC9030055; doi:10.3390/jpm12040538)
Supplement: Supplementary file 1 [file jpm-12-00538-s001.zip › jpm-1604417-supplementary.pdf]

Journal of Personalized Medicine

Supplementary Materials

Table S1. Missing variables, with more than 1% of missing values.

Variable	Count (%)
Support at home	17
PMS value	11
PMS Severity	9
Complication during pregnancy	5
Income	4
Complications during birth	3
Highest degree of education	3
Completed professional education	1
RLS	1
Birth-related psychological and physical traumas	1
RLS Value	1
Marital status	1
Stressful life events: Family	1
Family status	1
Stressful life events: Personal	1
Stressful life events	1
Total amount of children	1

Abbreviations: PMS, premenstrual syndrome; EPDS, Edinburgh Postnatal Depression Scale; MPAS, Maternal Postnatal Attachment Scale; SLE, stressful life events.

Table S2. Normality of EPDS values distributions.

EPDS	Cluster 1	Cluster 2	Cluster 3	Cluster 4	Cluster 5	Cluster 6
EPDS T0	--	--	--	+	--	--
EPDS T1	+	+	--	+	--	--
EPDS T2	--	--	--	--	--	+
EPDS T3	--	+	--	+	--	+
EPDS T4	--	+	--	+	--	--

Abbreviation: EPDS, Edinburgh Postnatal Depression Scale.+, normal distribution; --,not normal distribution.

Table S3. Significant differences of demographic features across patient groups.

Risk Factors	Values	Diagnosis			*p*-Value
		HC	PPD	AD	
Income	Less than 20,000/year	49 (9.9)	13 (16.4)	2 (1.1)	<0.001
	20,000–50,000/year	140 (30.9)	18 (32.7)	42 (46.7)	
	More than 50,000/year	264 (58.3)	24 (43.6)	46 (51.1)	
Familial psychiatric history	None	350 (77.3)	35 (63.6)	52 (57.8)	<0.001
	Yes	103 (22.7)	20 (36.4)	38 (42.2)	
PMS value	Mean (SD)	6.5 (6.4)	12.0 (8.0)	9.3 (6.8)	<0.001
Psychiatric diagnosis in previous pregnancy	None, no previous pregnancy	243 (53.6)	25 (45.5)	52 (57.8)	<0.001
	Yes	10 (2.2)	9 (16.4)	6 (6.7)	
	None, previous pregnancy	200 (44.2)	21 (38.2)	32 (35.6)	
Stressful life events	Mean (SD)	0.9 (1.3)	1.7 (1.8)	1.5 (1.6)	<0.001
Previous depression	None	418 (92.3)	33 (60.0)	64 (71.1)	<0.001
	Yes	35 (7.7)	22 (40.0)	26 (28.9)	
Baby blues	None	289 (63.8)	14 (25.5)	20 (22.2)	<0.001
	Yes	164 (36.2)	41 (74.5)	70 (77.8)	
Birth related psychological and physical traumas	None	410 (90.5)	43 (78.2)	62 (68.9)	<0.001
	Yes	43 (9.5)	12 (21.8)	28 (31.1)	

Abbreviations: HC, healthy controls; PPD, postpartum depression; AD, adjustment disorder; SD, standard deviation PMS, premenstrual syndrome; EPDS, Edinburgh Postnatal Depression Scale; MPAS, Maternal Postnatal Attachment Scale; SLE, stressful life events.

Table S4. Differences of features between HCs and AD patients, and between HCs and PPD patients.

Risk Factors	Values	Diagnosis		*p*-Value
		HC	AD	
Complications during birth	None	333 (73.5)	50 (55.6)	0.001
	Yes	120 (26.5)	40 (44.4)	
EPDS T0	Mean (SD)	4.3 (3.1)	9.6 (4.7)	<0.001
EPDS T1	Mean (SD)	4.5 (2.9)	10.4 (3.5)	<0.001
EPDS T2	Mean (SD)	3.3 (2.6)	7.9 (3.9)	<0.001
EPDS T3	Mean (SD)	2.9 (2.7)	5.8 (3.3)	<0.001
EPDS T4	Mean (SD)	2.5 (2.3)	5.3 (3.0)	<0.001
Familial psychiatric history	None	352 (77.7)	52 (57.8)	<0.001
	Yes	101 (22.3)	38 (42.2)	
MPAS T1	Mean (SD)	85.9 (5.1)	81.2 (7.3)	<0.001
MPAS T2	Mean (SD)	86.1 (5.2)	82.9 (5.9)	<0.001
MPAS T3	Mean (SD)	86.5 (5.1)	84.1 (5.6)	<0.001
MPAS T4	Mean (SD)	86.9 (4.8)	84.8 (5.7)	<0.001
PMS Severity	None	237 (52.3)	27 (30.0)	<0.001
	Moderate	163 (36.0)	40 (44.4)	
	Severe	53 (11.7)	23 (25.6)	
Stressful life events	Mean (SD)	0.9 (1.3)	1.5 (1.6)	<0.001
Support at home	Mean (SD)	1.8 (0.9)	2.3 (1.1	<0.001
Baby blues	None	290 (64.0)	20 (22.2)	<0.001
	Yes	163 (36.0)	70 (77.8)	
Birth related psychological and physical traumas	None	412 (90.9)	62 (68.9)	<0.001
	Yes	41 (9.1)	28 (31.1)	
Previous depression	None	419 (92.5)	64 (71.1)	<0.001
	Yes	34 (7.5)	26 (28.9)	
		Diagnosis		
		HC	PPD	
EPDS T0	Mean (SD)	4.3 (3.1)	8.5 (4.3)	<0.001
EPDS T1	Mean (SD)	4.5 (2.9)	12.0 (5.7)	<0.001

EPDS T2	Mean (SD)	3.3 (2.6)	12.5 (4.4)	<0.001
EPDS T3	Mean (SD)	2.9 (2.7)	12.4 (4.9)	<0.001
EPDS T4	Mean (SD)	2.5 (2.3)	13.0 (4.0)	<0.001
Family status	Single	6 (1.3)	6 (10.9)	<0.001
	With partner	447 (98.7)	49 (89.1)	
Income	Less than 20000/year	46 (10.2)	14 (25.5)	<0.001
	20000–50000/year	139 (30.7)	18 (32.7)	
	More than 50000/year	268 (59.2)	23 (41.8)	
MPAS T1	Mean (SD)	85.9 (5.1)	80.9 (7.3)	<0.001
MPAS T2	Mean (SD	86.1 (5.2)	78.6 (8.5)	<0.001
MPAS T3	Mean (SD)	86.5 (5.1)	79.0 (8.2)	<0.001
MPAS T4	Mean (SD)	86.9 (4.8)	79.5 (7.2)	<0.001
PMS Severity	None	237 (52.3)	12 (21.8)	<0.001
	Moderate	163 (36.0)	18 (32.7)	
	Severe	53 (11.7)	25 (45.5)	
Psychiatric diagnosis in previous pregnancy	None, no previouspregnancy	243 (53.6)	26 (47.3)	<0.001
	Yes	10 (2.2)	9 (16.4)	
	None, previous pregnancy	200 (44.2)	20 (36.4)	
Stressful life events	Mean (SD)	0.9 (1.3)	1.7 (1.8)	<0.001
Support at home	Mean (SD)	1.8 (0.9)	2.7 (1.5)	<0.001
Baby blues	None	290 (64.0)	13 (23.6)	<0.001
	Yes	163 (36.0)	42 (76.4)	
Previous depression	None	419 (92.5)	32 (58.2)	<0.001
	Yes	34 (7.5)	23 (41.8)	

Abbreviations: HC, healthy controls; PPD, postpartum depression; AD, adjustment disorder; SD, standard deviation PMS, premenstrual syndrome; EPDS, Edinburgh Postnatal Depression Scale; MPAS, Maternal Postnatal Attachment Scale; SLE, stressful life events.

Table S5. Differences of immediately after birth features across patient groups.

Risk Factors	Values	PPD	AD	*p*-Value
Baby blues	None	14 (25.5)	20 (22.2)	<0.001
	Yes	41 (74.5)	70 (77.8)	
EPDS T0	Mean (SD)	8.5 (4.3)	9.6 (4.7)	0.161
EPDS T1	Mean (SD)	12.0 (5.7)	10.4 (3.5)	0.044
EPDS T2	Mean (SD)	12.5 (4.4)	7.9 (3.9)	<0.001
EPDS T3	Mean (SD)	12.4 (4.9)	5.8 (3.3)	<0.001
EPDS T4	Mean (SD)	13.0 (4.0)	5.3 (3.0)	<0.001
Income	2	14 (25.5)	2 (2.2)	<0.001
	3	18 (32.7)	42 (46.7)	
	4	23 (41.8)	46 (51.1)	
MPAS T1	Mean (SD)	80.9 (7.3)	81.2 (7.3)	0.833
MPAS T2	Mean (SD)	78.6 (8.5)	82.9 (5.9)	<0.001
MPAS T3	Mean (SD)	79.0 (8.2)	84.1 (5.6)	<0.001
MPAS T4	Mean (SD)	79.5 (7.2)	84.8 (5.7)	<0.001

Abbreviations: HC, healthy controls; PPD, postpartum depression; AD, adjustment disorder; SD, standard deviation PMS, premenstrual syndrome; EPDS, Edinburgh Postnatal Depression Scale; MPAS, Maternal Postnatal Attachment Scale; SLE, stressful life events.

LCMM EPDS: Model Selection

Table S6. Best four models for LCMM of EPDS scores plus appropriate varying starting conditions.

Model Name	Nr Classes	AIC	BIC	SABIC	Entropy	LMR LRT	%class1	%class2	%class3	%class4	%class5	%class6
mQFQMQR4tf_nr	4	15002	15112	15033	0.70	Vs mQFQMQR3tf_nr $p < 0.001$ vs mQFQMQR5tf_nr $p = 1.000$	29.8	28.3	35.4	6.5	--	--
mQFQMQR4tf_nr_gridsearch	4	15002	15112	15033	0.70		35.4	28.3	29.8	6.5	--	--
mQFQR5tf_nr	5	15015	15112	15042	0.76	Vs mQFQR4tf_nr $p = 0.049$ vs mQFQR6tf_nr $p = 1.000$	3.5	24.1	28.1	34.8	9.5	--
mQFQR5tf_nr_gridsearch	5	15015	15112	15042	0.76		24.0	28.1	34.8	3.6	9.5	--
mQFQMLR5tf_nr	5	15022	15141	15055	0.73	Vs mQFQMLR4tf_nr $p < 0.001$ vs mQFQMLR6tf_nr $p = 1.000$	17.6	8.7	34.6	36.0	3.2	--
mQFQMLR5tf_nr_gridsearch	5	15022	15141	15055	0.73		8.7	3.2	34.6	35.9	17.6	--
mQFQM6ff_nr	6	15083	15192	15113	0.82	Vs mQFQM5ff_nr $p < 0.001$ vs mQFQM7ff_nr $p = 1.000$	9.5	2.8	5.2	7.6	71.5	3.3
mQFQM6ff_nr_gridsearch	6	15083	15192	15113	0.82		7.6	5.2	9.6	71.6	3.3	2.8

The trajectories of the calculated models are provided in Figure S1. Based on information criteria, entropy and trajectory shapes, we focus on the two models: the five-class model solution with quadratic dependence of fixed and mixed effects, and linear dependence of random effects (mQFQMLR5tf_nr); and the six-class model solution with quadratic dependence of fixed and mixed effects, and constant dependence of random effects (mQFQM6ff_nr). Although AIC, BIC and SABIC results are lower in four-class solution mQFQMLR5tf_nr and mQFQM6ff_nr possess higher entropy. Upon closer inspection, the similarities between the shapes of trajectories are obvious. However, the five cluster solution differ only in magnitude, and we expect difference in the shape due to different diagnoses. Abbreviations: AIC, the Akaike information criterion; BIC, Bayesian information criterion; SABIC, the sample-adjusted Bayesian information criterion; LMR LRT, the Lo–Mendell–Rubin likelihood test. --,not normal distribution.

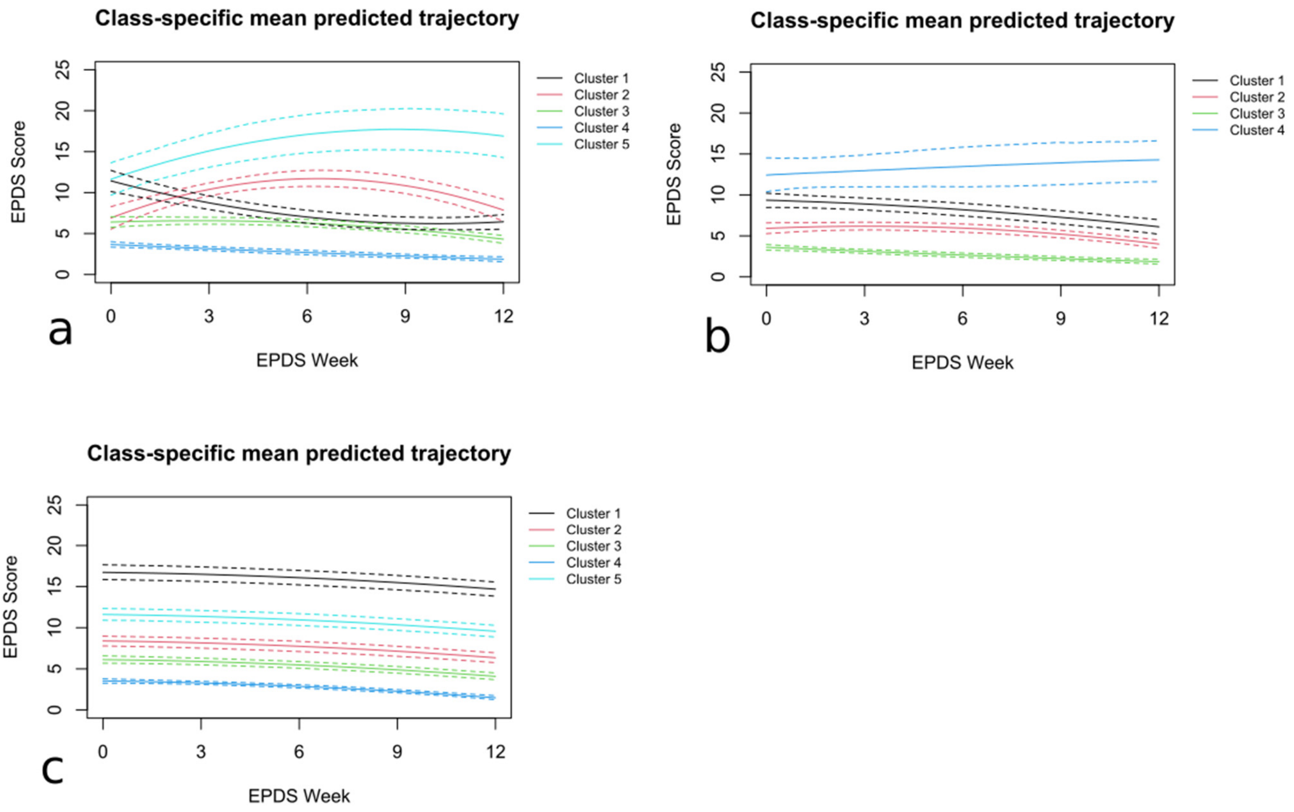

Figure S1. Plots of EPDS clusters for models from the table S6: **(a)** mQFQMQR4tf_nr, **(b)** mQFQR5tf_nr, **(c)** mQFQMLR5tf_nr. EPDS, Edinburgh Postnatal Depression Scale.

Results LCMM EPDS: Clusters

Table S7. Total association between prenatal features in EPDS clusters.

Risk Factors	Values	Clusters						*p*-Value
		1	2	3	4	5	6	
EPDS T0	Mean (SD)	10.5 (2.2)	10.8 (3.1)	14.4 (3.7)	7.0 (2.8)	3.9 (2.7)	3.3 (2.2)	<0.001
EPDS T1	Mean (SD)	10.2 (3.4)	16.4 (6.3)	8.9 (3.6)	9.5 (3.4)	4.3 (2.9)	10.9 (3.6)	<0.001
EPDS T2	Mean (SD)	8.1 (2.5)	16.8 (3.2)	5.1 (4.4)	10.2 (2.7)	3.0 (2.4)	11.6 (2.6)	<0.001
EPDS T3	Mean (SD)	6.3 (2.7)	17.5 (2.9)	3.7 (3.1)	10.4 (2.8)	2.6 (2.4)	8.6 (3.0)	<0.001
EPDS T4	Mean (SD)	5.3 (2.5)	17.6 (2.7)	3.6 (3.2)	10.3 (2.6)	2.4 (2.4)	4.6 (2.6)	<0.001
Familial psychiatric history	None	32 (56.1)	8 (47.1)	19 (61.3)	26 (57.8)	336 (78.5)	17 (85.0)	<0.001
	Yes	25 (43.9)	9 (52.9)	12 (38.7)	19 (42.2)	92 (21.5)	3 (15.0)	
Family status	Single parent	0 (0.0)	4 (23.5)	1 (3.2)	3 (6.7)	6 (1.4)	0 (0.0)	<0.001
	With partner	57 (100.0)	13 (76.5)	30 (96.8)	42 (93.3)	422 (98.6)	20 (100.0)	
Income	Less than 20,000/year	0 (0.0)	5 (29.4)	1 (3.2)	0 (0.0)	3 (0.7)	0 (0.0)	<0.001
	20,000–50,000/year	2 (3.5)	4 (23.5)	1 (3.2)	4 (8.9)	40 (9.3)	2 (10.0)	
	More than 50,000/year	26 (45.6)	4 (23.5)	18 (58.1)	15 (33.3)	129 (30.1)	7 (35.0)	
MPAS T1	Mean (SD)	81.2 (7.2)	80.1 (8.4)	84.9 (6.3)	81.4 (7.0)	85.9 (5.1)	81.2 (7.5)	<0.001
MPAS T2	Mean (SD)	82.8 (6.0)	76.6 (8.8)	86.4 (4.4)	80.1 (8.0)	86.1 (5.2)	82.2 (6.3)	<0.001
MPAS T3	Mean (SD)	83.7 (5.8)	74.7 (9.0)	87.8 (3.6)	81.6 (6.7)	86.5 (5.1)	83.3 (7.5)	<0.001
MPAS T4	Mean (SD)	84.6 (6.0)	77.8 (8.5)	87.7 (3.9)	81.3 (6.3)	86.8 (5.0)	84.8 (4.9)	<0.001
PMS Severity	None	19 (33.3)	1 (5.9)	11 (35.5)	11 (24.4)	229 (53.5)	5 (25.0)	<0.001
	Moderate	23 (40.4)	7 (41.2)	16 (51.6)	15 (33.3)	153 (35.7)	7 (35.0)	

	Severe	15 (26.3)	9 (52.9)	4 (12.9)	19 (42.2)	46 (10.7)	8 (40.0)	
Psychiatric diagnosis in previous pregnancy	None, no previous pregnancy	30 (52.6)	5 (29.4)	22 (71.0)	26 (57.8)	226 (52.8)	12 (60.0)	<0.001
	Yes	7 (12.3)	8 (47.1)	2 (6.5)	1 (2.2)	6 (1.4)	1 (5.0)	
	None, previous pregnancy	20 (35.1)	4 (23.5)	7 (22.6)	18 (40.0)	196 (45.8)	7 (35.0)	
Relocation to the other ward	None	29 (50.9)	13 (76.5)	17 (54.8)	34 (75.6)	327 (76.4)	16 (80.0)	<0.001
	Yes	28 (49.1)	4 (23.5)	14 (45.2)	11 (24.4)	101 (23.6)	4 (20.0)	
Stressful life events Support at home	0	16 (28.1)	2 (11.8)	9 (29.0)	20 (44.4)	239 (55.8)	14 (70.0)	<0.001
	1	23 (40.4)	4 (23.5)	8 (25.8)	5 (11.1)	98 (22.9)	3 (15.0)	
	2	8 (14.0)	5 (29.4)	4 (12.9)	8 (17.8)	45 (10.5)	2 (10.0)	
	3	7 (12.3)	3 (17.6)	4 (12.9)	7 (15.6	24 (5.6)	1 (5.0)	
	4	3 (5.3)	0 (0.0)	3 (9.7)	1 (2.2)	12 (2.8)	0 (0.0)	
	5	0 (0.0)	2 (11.8)	1 (3.2)	2 (4.4)	3 (0.7)	0 (0.0)	
	6	0 (0.0)	1 (5.9)	2 (6.5)	0 (0.0)	5 (1.2)	0 (0.0)	
	7	0 (0.0)	0 (0.0)	0 (0.0)	1 (2.2)	1 (0.2)	0 (0.0)	
	8	0 (0.0)	0 (0.0)	0 (0.0)	1 (2.2)	0 (0.0)	0 (0.0)	
	9	0 (0.0)	0 (0.0)	0 (0.0)	0 (0.0)	1 (0.2)	0 (0.0)	
Support at home	1	21 (36.8)	2 (11.8)	16 (51.6)	8 (17.8)	184 (43)	8 (40.0)	<0.001
	2	14 (24.6)	6 (35.3)	4 (12.9)	19 (42.2)	173 (40.4)	7 (35.0)	
	3	18 (31.6)	2 (11.8)	6 (19.4)	8 (17.8)	54 (12.6)	2 (10.0)	
	4	3 (5.3)	3 (17.6)	4 (12.9)	6 (13.3)	12 (2.8)	2 (10.0)	
	5	1 (1.8	3 (17.6)	1 (3.2)	3 (6.7)	4 (0.9)	1 (5.0)	
	6	0 (0.0)	1 (5.9)	0 (0.0)	1 (2.2)	1 (0.2)	0 (0.0)	
Baby blues	None	17 (29.8)	3 (17.6	11 (35.5)	12 (26.7)	277 (64.7)	3 (15.0)	<0.001
	Yes	40 (70.2)	14 (82.4)	20 (64.5)	33 (73.3)	151 (35.3)	17 (85.0)	
Birth related psychological and physical traumas	None	40 (70.2)	15 (88.2)	24 (77.4)	36 (80.0)	390 (91.1)	12 (60.0)	<0.001
	Yes	17 (29.8)	2 (11.8)	7 (22.6)	9 (20.0)	38 (8.9)	8 (40.0)	
Previous depression	None	40 (70.2)	10 (58.8)	21 (67.7)	28 (62.2)	400 (93.5)	16 (80.0)	<0.001
	Yes	17 (29.8)	7 (41.2)	10 (32.3)	17 (37.8)	28 (6.5)	4 (20.0)	
Target	HC	17 (29.8)	0 (0.0)	9 (29.0)	7 (15.6)	416 (97.2)	4 (20.0)	<0.001
	PPD	2 (3.5)	17 (100.0)	1 (3.2)	29 (64.4)	3 (0.7)	3 (15.0)	
	AD	38 (66.7)	0 (0.0)	21 (67.7)	9 (20.0)	9 (2.1)	13 (65.0)	

Abbreviations: HC, healthy controls; PPD, postpartum depression; AD, adjustment disorder; SD, standard deviation PMS, premenstrual syndrome; EPDS, Edinburgh Postnatal Depression Scale; MPAS, Maternal Postnatal Attachment Scale; SLE, stressful life events.

Table S8. Significant differences between clusters 1 vs 5.

Risk Factors	Values	Clusters		*p*-Value
		1	5	
Familial psychiatric history	None	32 (56.1)	336 (78.5)	<0.001
	Yes	25 (43.9)	92 (21.5)	
MPAS T1	Mean (SD)	81.2 (7.2)	85.9 (5.1)	<0.001
MPAS T2	Mean (SD)	82.8 (6.0)	86.1 (5.2)	<0.001
MPAS T3	Mean (SD)	83.7 (5.8)	86.5 (5.1)	<0.001
Psychiatric diagnosis in previous pregnancy	None, no previous pregnancy	30 (52.6)	226 (52.8)	<0.001
	Yes	7 (12.3)	6 (1.4)	
	None, previous pregnancy	20 (35.1)	196 (45.8)	
Relocation to the other ward	None	29 (50.9)	327 (76.4)	<0.001
	Yes	28 (49.1)	101 (23.6)	
Baby blues	None	17 (29.8)	277 (64.7)	<0.001
	Yes	40 (70.2)	151 (35.3)	
	None	40 (70.2)	390 (91.1)	<0.001

Birth related psychological and physical traumas	Yes	17 (29.8)	38 (8.9)	
Previous depression	None	40 (70.2)	400 (93.5)	<0.001
	Yes	17 (29.8)	28 (6.5)	
Target	HC	17 (29.8)	416 (97.2)	<0.001
	PPD	2 (3.5)	3 (0.7)	
	AD	38 (66.7)	9 (2.1)	

Abbreviations: HC, healthy controls; PPD, postpartum depression; AD, adjustment disorder; SD, standard deviation PMS, premenstrual syndrome; EPDS, Edinburgh Postnatal Depression Scale; MPAS, Maternal Postnatal Attachment Scale; SLE, stressful life events.

Table S9. Significant differences between clusters 2 vs 5.

**Risk Factors**	**Values**	**Clusters**		***p*-Value**
		**2**	**5**	
Family status	None	4 (23.5)	6 (1.4)	<0.001
	Yes	13 (76.5)	422 (98.6)	
Income	Less than 20,000/year	9 (52.9)	43 (10.1)	<0.001
	20,000–50,000/year	4 (23.5)	129 (30.1)	
	More than 50,000/year	4 (23.5)	256 (59.8)	
MPAS T1	Mean (SD)	80.1 (8.4)	85.9 (5.1)	<0.001
MPAS T2	Mean (SD)	76.6 (8.8)	86.1 (5.2)	<0.001
MPAS T3	Mean (SD)	74.7 (9.0)	86.5 (5.1)	<0.001
MPAS T4	Mean (SD)	77.8 (8.5)	86.8 (5.0)	<0.001
PMS Severity	0	1 (5.9)	229 (53.5)	<0.001
	1	7 (41.2)	153 (35.7)	
	2	9 (52.9)	46 (10.7)	
Psychiatric diagnosis in previous pregnancy	None, no previous pregnancy	5 (29.4)	226 (52.8)	<0.001
	Yes	8 (47.1)	6 (1.4)	
	None, previous pregnancy	4 (23.5)	196 (45.8)	
Stressful life events	0	2 (11.8)	239 (55.8)	<0.001
	1	4 (23.5)	98 (22.9)	
	2	5 (29.4)	45 (10.5)	
	3	3 (17.6)	24 (5.6)	
	4	0 (0.0)	12 (2.8)	
	5	2 (11.8)	3 (0.7)	
	6	1 (5.9)	5 (1.2)	
	7	0 (0.0)	1 (0.2)	
	8	0 (0.0)	0 (0.0)	
	9	0 (0.0)	1 (0.2)	
Support at home	1	2 (11.8)	184 (43.0)	<0.001
	2	6 (35.3)	173 (40.4)	
	3	2 (11.8)	54 (12.6)	
	4	3 (17.6)	12 (2.8)	
	5	3 (17.6)	4 (0.9)	
	6	1 (5.9)	1 (0.2)	
Baby blues	None	3 (17.6)	277 (64.7)	<0.001
	Yes	14 (82.4)	151 (35.3)	
Previous depression	None	10 (58.8)	400 (93.5)	<0.001
	Yes	7 (41.2)	28 (6.5)	
Target	HC	0 (0.0)	416 (97.2)	<0.001
	PPD	17 (100.0)	3 (0.7)	
	AD	0 (0.0)	9 (2.1)	

Abbreviations: HC, healthy controls; PPD, postpartum depression; AD, adjustment disorder; SD, standard deviation PMS, premenstrual syndrome; EPDS, Edinburgh Postnatal Depression Scale; MPAS, Maternal Postnatal Attachment Scale; SLE, stressful life events.

Table S10. Significant differences between clusters 3 vs 5.

Risk Factors	Values	Clusters		p-Value
		3	5	
Previous depression	None	21 (67.7)	400 (93.5)	<0.001
	Yes	10 (32.3)	28 (6.5)	
Target	HC	9 (29.0)	416 (97.2)	<0.001
	PPD	1 (3.2)	3 (0.7)	
	AD	21 (67.7)	9 (2.1)	

Abbreviations: HC, healthy controls; PPD, postpartum depression; AD, adjustment disorder; SD, standard deviation PMS, premenstrual syndrome; EPDS, Edinburgh Postnatal Depression Scale; MPAS, Maternal Postnatal Attachment Scale; SLE, stressful life events.

Table S11. Significant differences between clusters 4 vs 5.

Risk Factors	Values	Clusters		p-Value
		4	5	
MPAS T1	Mean (SD)	81.4 (7.0)	85.9 (5.1)	<0.001
MPAS T2	Mean (SD)	80.1 (8.0)	86.1 (5.2)	<0.001
MPAS T3	Mean (SD)	81.6 (6.7)	86.5 (5.1)	<0.001
MPAS T4	Mean (SD)	81.3 (6.3)	86.8 (5.0)	<0.001
PMS Severity	None	11 (24.4)	229 (53.5)	<0.001
	Moderate	15 (33.3)	153 (35.7)	
	Severe	19 (42.2)	46 (10.7)	
Stressful life events	0	20 (44.4)	239 (55.8)	<0.001
	1	5 (11.1)	98 (22.9)	
	2	8 (17.8)	45 (10.5)	
	3	7 (15.6)	24 (5.6)	
	4	1 (2.2)	12 (2.8)	
	5	2 (4.4)	3 (0.7)	
	6	0 (0.0)	5 (1.2)	
	7	1 (2.2)	1 (0.2)	
	8	1 (2.2)	0 (0.0)	
	9	0 (0.0)	1 (0.2)	
Support at home	1	8 (17.8)	182 (43.0)	<0.001
	2	19 (42.2)	173 (40.4)	
	3	8 (17.8)	54 (12.6)	
	4	6 (13.3)	12 (2.8)	
	5	3 (6.7)	4 (0.9)	
	6	1 (2.2)	1 (0.2)	
Baby blues	None	12 (26.7)	277 (64.7)	<0.001
	Yes	33 (73.3)	151 (35.3)	
Previous depression	None	28 (62.2)	400 (93.5)	<0.001
	Yes	17 (37.8)	28 (6.5)	
Target	HC	7 (15.6)	416 (97.2)	<0.001
	PPD	29 (64.4)	3 (0.7)	
	AD	9 (20.0)	9 (2.1)	

Abbreviations: HC, healthy controls; PPD, postpartum depression; AD, adjustment disorder; SD, standard deviation PMS, premenstrual syndrome; EPDS, Edinburgh Postnatal Depression Scale; MPAS, Maternal Postnatal Attachment Scale; SLE, stressful life events.

Table S12. Significant differences between clusters 6 vs 5.

Risk Factors	Values	Clusters		*p*-Value
		5	6	
MPAS T1	Mean (SD)	85.9 (5.1)	81.2 (7.5)	<0.001
PMS Severity	None	229 (53.5)	5 (25.0)	<0.001
	Moderate	153 (35.7)	7 (35.0)	
	Severe	46 (10.7)	8 (40.0)	
Baby blues	None	277 (64.7)	3 (15.0)	<0.001
	Yes	151 (35.3)	17 (85.0)	
Birth related psychological and physical traumas	None	390 (91.1)	12 (60.0)	<0.001
	Yes	38 (8.9)	8 (40.0)	
Target	HC	416 (97.2)	4 (20.0)	<0.001
	PPD	3 (0.7)	3 (15.0)	
	AD	9 (2.1)	13 (65.0)	

Abbreviations: HC, healthy controls; PPD, postpartum depression; AD, adjustment disorder; SD, standard deviation PMS, premenstrual syndrome; EPDS, Edinburgh Postnatal Depression Scale; MPAS, Maternal Postnatal Attachment Scale; SLE, stressful life events.

Table S13. Significant differences between clusters 1 vs 3 vs 6.

Risk Factors	Values	Clusters			*p*-Value
		1	3	6	
EPDS T0	Mean (SD)	10.5 (2.2)	14.4 (3.7)	3.3 (2.2)	<0.001
EPDS T2	Mean (SD)	8.1 (2.5)	5.1 (4.4)	11.6 (2.6)	<0.001
EPDS T3	Mean (SD)	6.3 (2.7)	3.7 (3.1)	8.6 (3.0)	<0.001

Abbreviations: SD, standard deviation; EPDS, Edinburgh Postnatal Depression Scale.

Table S14. Significant differences between clusters 2 vs 4 and comparison between PPD in clusters 2 vs 4.

Risk Factors	Values	Clusters		*p*-Value
		2	4	
EPDS T0	Mean (SD)	10.8 (3.1)	7.0 (2.8)	<0.001
EPDS T1	Mean (SD)	16.4 (6.3)	9.5 (3.4)	<0.001
EPDS T2	Mean (SD)	16.8 (3.2)	10.2 (2.7)	<0.001
EPDS T3	Mean (SD)	17.5 (2.9)	10.4 (2.8)	<0.001
EPDS T4	Mean (SD)	17.6 (2.7)	10.3 (2.6)	<0.001
Income	Less than 20,000/year	9 (53.0)	4 (8.9)	<0.001
	20,000–50,000/year	4 (23.5)	15 (33.3)	
	More than 50,000/year	4 (23.5)	26 (57.8)	
Psychiatric diagnosis in previous pregnancy	None, no previous pregnancy	5 (29.4)	26 (57.8)	<0.001
	Yes	8 (47.1)	1 (2.2)	
	None, previous pregnancy	4 (23.5)	18 (40.0)	
Target	HC	0 (0.0)	7 (15.6)	0.017
	PPD	17 (100.0)	29 (64.4)	
	AD	0 (0.0)	9 (20.0)	
		**PPD in Clusters**		
		2	4	
EPDS_T0	Mean (SD)	10.8 (3.1)	7.4 (3.0)	0.001
EPDS_T1	Mean (SD)	16.4 (6.3)	9.9 (3.3)	<0.001
EPDS_T2	Mean (SD)	16.8 (3.2)	11.2 (2.2)	<0.001
EPDS_T3	Mean (SD)	17.5 (2.9)	10.4 (2.9)	<0.001
EPDS_T4	Mean (SD)	17.6 (2.7)	11.3 (2.2)	<0.001
Psychiatric diagnosis in previous pregnancy	None, no previous pregnancy	5 (29.4)	16 (55.2)	0.002
	Yes	8 (47.1)	1 (3.4)	
	None, previous pregnancy	4 (23.5)	12 (41.4)	

Abbreviations: HC, healthy controls; PPD, postpartum depression; AD, adjustment disorder; SD, standard deviation PMS, premenstrual syndrome; EPDS, Edinburgh Postnatal Depression Scale; MPAS, Maternal Postnatal Attachment Scale; SLE, stressful life events.

Results multLCMM MPAS + EPDS: Model Selection

The best two models for multlcmm MPAS with lowest values of SABIC and AIC and high entropy were considered and are provided in Table S15. Both 5 and 6 class models were unstable under varying starting conditions. Again, the Lo–Mendell–Rubin likelihood (LMR LRT) tests were performed.

Table S15. Total association between prenatal features in MPAS-EPDS clusters.

Model Name	Nr Classes	AIC	BIC	SABIC	Entropy	LMR LRT	%class1	%class2	%class3	%class4	%class5	%class6
multQFQMLR4_tf	4	26401	26511	26432	0.72	Vs mQFQMLR3tf_nr *p* < 0.001 vs mQFQMLR5tf_nr *p* = 0.027	30.9	46.5	11.4	11.2	--	--
multQFQMLR4_tf_gridsearch	4	26401	26511	26432	0.72		46.5	30.9	11.4	11.2	--	--
multQFQM6_tf	6	26431	26576	26471	0.74	Vs mQFQM5tf_nr *p* < 0.001 vs mQFQM7tf_nr *p* = 1.000	30.1	48.3	6.0	5.1	6.3	4.2
multQFQM6_tf_gridsearch	6	26414	26559	26454	0.76		46.7	7.7	4.0	1.0	29.6	11.0

Abbreviations: AIC, the Akaike information criterion; BIC, Bayesian information criterion; SABIC, the sample-adjusted Bayesian information criterion; LMR LRT, the Lo–Mendell–Rubin likelihood test. --,not normal distribution.

Table S16. Significant differences between all MPAS/EPDS clusters.

Risk Factors	Values	Clusters 1	Clusters 2	Clusters 3	Clusters 4	*p*-Value
EPDS T0	Mean (SD)	5.7 (3.6)	3.1 (2.8)	8.7 (4.5)	7.8 (4.6)	<0.001
EPDS T1	Mean (SD)	6.3 (2.3)	2.1 (1.7)	13.1 (4.4)	9.1 (4.2)	<0.001
EPDS T2	Mean (SD)	4.8 (2.3)	1.3 (1.3)	7.9 (4.7)	11.8 (4.0)	<0.001
EPDS T3	Mean (SD)	4.2 (2.3)	0.9 (1.1)	6.4 (4.7)	11.6 (4.0)	<0.001
EPDS T4	Mean (SD)	3.5 (2.1)	0.8 (1.0)	8.4 (5.0)	9.1 (4.8)	<0.001
Familial psychiatric history	None	206 (74.1)	152 (82.2)	39 (57.4)	41 (61.2)	<0.001
	Yes	72 (25.9)	33 (17.8)	29 (42.6)	26 (38.8)	
MPAS T1	Mean (SD)	84.3 (5.2)	88.2 (3.9)	79.7 (8.1)	82.1 (6.6)	<0.001
MPAS T2	Mean (SD)	84.6 (5.3)	88.4 (3.9)	82.5 (6.5)	79.3 (8.1)	<0.001
MPAS T3	Mean (SD)	85.2 (4.9)	88.9 (3.8)	83.3 (5.7)	79.4 (8.6)	<0.001
MPAS T4	Mean (SD)	85.7 (4.8)	89.0 (3.7)	83.1 (6.6)	81.1 (7.2)	<0.001
PMS Severity	None	116 (41.7)	128 (69.2)	13 (19.1)	19 (28.4)	<0.001
	Moderate	119 (42.8)	49 (26.5)	33 (48.5)	20 (29.9)	
	Severe	43 (15.5)	8 (4.3)	22 (32.4)	28 (41.8)	
Psychiatric diagnosis in previous pregnancy	None, noprevious pregnancy	157 (56.5)	93 (50.3)	43 (63.2)	28 (41.8)	<0.001
	Yes	9 (3.2)	2 (1.1)	5 (7.4)	9 (13.4)	
	None, previous pregnancy	112 (40.3)	90 (48.6)	20 (29.4)	30 (44.8)	
Support at home	1	106 (38.1)	97 (52.5)	23 (33.8)	13 (19.4)	<0.001
	2	117 (42.1)	64 (34.6)	16 (23.5)	26 (38.8)	
	3	43 (15.5)	18 (9.7)	15 (22.1)	14 (20.9)	
	4	9 (3.2)	4 (2.2)	9 (13.2)	8 (11.9)	
	5	3 (1.1)	1 (0.5)	4 (5.9)	5 (7.5)	
	6	0 (0.0)	1 (0.5)	1 (1.5)	1 (1.5)	

Baby blues	None	149 (53.6)	145 (78.4)	9 (13.2)	20 (29.9)	<0.001
	Yes	129 (46.4)	40 (21.6)	59 (86.8)	47 (70.1)	
Birth related psychological and physical traumas	None	241 (86.7)	175 (94.6)	49 (72.1)	52 (77.6)	<0.001
	Yes	37 (13.3)	10 (5.4)	19 (27.9)	15 (22.4)	
Previous depression	None	243 (87.4)	176 (95.1)	50 (73.5)	46 (68.7)	<0.001
	Yes	35 (12.6)	9 (4.9)	18 (26.5)	21 (31.3)	
Target	HC	248 (89.2)	183 (98.9)	8 (11.8)	14 (20.9)	<0.001
	PPD	0 (0.0)	0 (0.0)	21 (30.9)	34 (50.7)	
	AD	30 (10.8)	2 (1.1)	39 (57.4)	19 (28.4)	

Abbreviations: HC, healthy controls; PPD, postpartum depression; AD, adjustment disorder; SD, standard deviation PMS, premenstrual syndrome; EPDS, Edinburgh Postnatal Depression Scale; MPAS, Maternal Postnatal Attachment Scale; SLE, stressful life events.

Table S17. Significant differences between clusters 1 vs 2.

**Risk Factors**	**Values**	**Clusters**		***p*-Value**
		**1**	**2**	
EPDS T0	Mean (SD)	5.7 (3.6)	3.1 (2.8)	<0.001
EPDS T1	Mean (SD)	6.3 (2.3)	2.1 (1.7)	<0.001
EPDS T2	Mean (SD)	4.8 (2.3)	1.3 (1.3)	<0.001
EPDS T3	Mean (SD)	4.2 (2.3)	0.9 (1.1)	<0.001
EPDS T4	Mean (SD)	3.5 (2.1)	0.8 (1.0)	<0.001
MPAS T1	Mean (SD)	84.3 (5.2)	88.2 (3.9)	<0.001
MPAS T2	Mean (SD)	84.6 (5.3)	88.4 (3.9)	<0.001
MPAS T3	Mean (SD)	85.2 (4.9)	88.9 (3.8)	<0.001
MPAS T4	Mean (SD)	85.7 (4.8)	89.0 (3.7)	<0.001
PMS Severity	None	116 (41.7)	128 (69.2)	<0.001
	Moderate	119 (42.8)	49 (26.5)	
	Severe	43 (15.5)	8 (4.3)	
Baby blues	No	149 (53.6)	145 (78.4)	<0.001
	Yes	129 (46.4)	40 (21.6)	
Target	HC	248 (89.2)	183 (98.9)	<0.001
	PPD	0 (0.0)	0 (0.0)	
	AD	30 (10.8)	2 (1.1)	

Abbreviations: HC, healthy controls; PPD, postpartum depression; AD, adjustment disorder; SD, standard deviation PMS, premenstrual syndrome; EPDS, Edinburgh Postnatal Depression Scale; MPAS, Maternal Postnatal Attachment Scale; SLE, stressful life events.

Table S18. Significant differences between clusters 2 vs 3.

**Risk Factors**	**Values**	**Clusters**		***p*-Value**
		**2**	**3**	
EPDS T0	Mean (SD)	3.1 (2.8)	8.7 (4.5)	<0.001
EPDS T1	Mean (SD)	2.1 (1.7)	13.1 (4.4)	<0.001
EPDS T2	Mean (SD)	1.3 (1.3)	7.9 (4.7)	<0.001
EPDS T3	Mean (SD)	0.9 (1.1)	6.4 (4.7)	<0.001
EPDS T4	Mean (SD)	0.8 (1.0)	8.4 (5.0)	<0.001
Familial psychiatric history	None	152 (82.2)	39 (57.4)	<0.001
	Yes	33 (17.8)	29 (42.6)	
MPAS T1	Mean (SD)	88.2 (3.9)	79.7 (8.1)	<0.001
MPAS T2	Mean (SD)	88.4 (3.9)	82.5 (6.5)	<0.001
MPAS T3	Mean (SD)	88.9 (3.8)	83.3 (5.7)	<0.001
MPAS T4	Mean (SD)	89.0 (3.7)	83.1 (6.6)	<0.001
PMS Severity	0	128 (69.2)	13 (19.1)	<0.001
	1	49 (26.5)	33 (48.5)	
	2	8 (4.3)	22 (32.4)	
Support at home	1	97 (52.5)	23 (33.8)	<0.001

	2	64 (34.6)	16 (23.5)	
	3	18 (9.7)	15 (22.1)	
	4	4 (2.2)	9 (13.2)	
	5	1 (0.5)	4 (5.9)	
	6	1 (0.5)	1 (1.5)	
Baby blues	None	145 (78.4)	9 (13.2)	<0.001
	Yes	40 (21.6)	59 (86.8)	
Birth related psychological and physical traumas	None	175 (94.6)	49 (72.1)	<0.001
	Yes	10 (5.4)	19 (27.9)	
Previous depression	None	176 (95.1)	50 (73.5)	<0.001
	Yes	9 (4.9)	18 (26.5)	
Target	HC	183 (98.9)	8 (11.8)	<0.001
	PPD	0 (0.0)	21 (30.9)	
	AD	2 (1.1)	39 (57.4)	

Abbreviations: HC, healthy controls; PPD, postpartum depression; AD, adjustment disorder; SD, standard deviation PMS, premenstrual syndrome; EPDS, Edinburgh Postnatal Depression Scale; MPAS, Maternal Postnatal Attachment Scale; SLE, stressful life events.

Table S19. Significant differences between clusters 2 vs 4.

Risk Factors	Values	Clusters		*p*-Value
		2	4	
EPDS T0	Mean (SD)	3.1 (2.8)	7.8 (4.6)	<0.001
EPDS T1	Mean (SD)	2.1 (1.7)	9.1 (4.2)	<0.001
EPDS T2	Mean (SD)	1.3 (1.3)	11.8 (4.0)	<0.001
EPDS T3	Mean (SD)	0.9 (1.1)	11.6 (4.0)	<0.001
EPDS T4	Mean (SD)	0.8 (1.0)	9.1 (4.8)	<0.001
MPAS T1	Mean (SD)	88.2 (3.9)	82.1 (6.6)	<0.001
MPAS T2	Mean (SD)	88.4 (3.9)	79.3 (8.1)	<0.001
MPAS T3	Mean (SD)	88.9 (3.8)	79.4 (8.6)	<0.001
MPAS T4	Mean (SD)	89.0 (3.7)	81.1 (7.2)	<0.001
PMS Severity	None	128 (69.2)	19 (28.4)	<0.001
	Moderate	49 (26.5)	20 (29.9)	
	Severe	8 (4.3)	28 (41.8)	
Psychiatric diagnosis in previous pregnancy	None, no previous pregnancy	93 (50.3)	28 (41.8)	<0.001
	Yes	2 (1.1)	9 (13.4)	
	None, previous pregnancy	90 (48.6)	30 (44.8)	
Stressful life events	0	111 (60.0)	29 (43.3)	<0.001
	1	44 (23.8)	7 (10.4)	
	2	16 (8.6)	14 (20.9)	
	3	8 (4.3)	8 (11.9)	
	4	5 (2.7)	3 (4.5)	
	5	0 (0.0)	4 (6.0)	
	6	1 (0.5)	1 (1.5)	
	7	0 (0.0)	1 (1.5)	
	8	0 (0.0)	0 (0.0)	
	9	0 (0.0)	0 (0.0)	
Support at home	1	97 (52.5)	13 (19.4)	<0.001
	2	64 (34.6)	26 (38.8)	
	3	18 (9.7)	14 (20.9)	
	4	4 (2.2)	8 (11.9)	
	5	1 (0.5)	5 (7.5)	
	6	1 (0.5)	1 (1.5)	
Baby blues	None	145 (78.4)	20 (29.9)	<0.001
	Yes	40 (21.6)	47 (70.1)	

Birth related psychological and physical traumas	None	175 (94.6)	52 (77.6)	<0.001
	Yes	10 (5.4)	15 (22.4)	
Previous depression	None	176 (95.1)	46 (68.7)	<0.001
	Yes	9 (4.9)	21 (31.3)	
Target	HC	183 (98.9)	14 (20.9)	<0.001
	PPD	0 (0.0)	34 (50.7)	
	AD	2 (1.1)	19 (28.4)	

Abbreviations: HC, healthy controls; PPD, postpartum depression; AD, adjustment disorder; SD, standard deviation PMS, premenstrual syndrome; EPDS, Edinburgh Postnatal Depression Scale; MPAS, Maternal Postnatal Attachment Scale; SLE, stressful life events.

Table S20. Significant differences between clusters 3 vs 4.

Risk Factors	Values	Clusters		*p*-Value
		3	4	
EPDS_T1	Mean (SD)	13.1 (4.4)	9.1 (4.2)	<0.001
EPDS_T2	Mean (SD)	7.9 (4.7)	11.8 (4.0)	<0.001
EPDS_T3	Mean (SD)	6.4 (4.7)	11.6 (4.0)	<0.001

Abbreviations: SD, standard deviation; EPDS, Edinburgh Postnatal Depression Scale.

Table S21. Significant differences between HCs in cluster 1 and baseline (cluster 2).

Risk Factors	Values	Clusters		*p*-Value
		1	2	
EPDS_T0	Mean (SD)	5.1 (3.1)	2.9 (2.6)	<0.001
EPDS_T1	Mean (SD)	6.0 (2.1)	2.1 (1.7)	<0.001
EPDS_T2	Mean (SD)	4.5 (2.1)	1.3 (1.3)	<0.001
EPDS_T3	Mean (SD)	4.1 (2.3)	0.9 (1.1)	<0.001
EPDS_T4	Mean (SD)	3.4 (2.1)	0.8 (1.0)	<0.001
MPAS_T1	Mean (SD)	84.5 (5.3)	88.2 (3.9)	<0.001
MPAS_T2	Mean (SD)	84.7 (5.3)	88.3 (3.9)	<0.001
MPAS_T3	Mean (SD)	85.1 (4.9)	88.9 (3.9)	<0.001
MPAS_T4	Mean (SD)	85.7 (4.8)	89.0 (3.8)	<0.001
PMS Severity	None	104 (41.9)	128 (69.9)	<0.001
	Moderate	106 (42.7)	47 (25.7)	
	Severe	38 (15.3)	8 (4.4)	
Baby blues	None	137 (55.2)	144 (78.7)	<0.001
	Yes	111 (44.8)	39 (21.3)	

Abbreviations: HC, healthy controls; PPD, postpartum depression; AD, adjustment disorder; SD, standard deviation PMS, premenstrual syndrome; EPDS, Edinburgh Postnatal Depression Scale; MPAS, Maternal Postnatal Attachment Scale; SLE, stressful life events.
